# Clinical significance of preoperative CALLY index for prognostication in patients with esophageal squamous cell carcinoma undergoing surgery

**DOI:** 10.1038/s41598-023-51109-w

**Published:** 2024-01-06

**Authors:** Jifeng Feng, Liang Wang, Xun Yang, Qixun Chen

**Affiliations:** 1 Department of Thoracic Surgery, Zhejiang Cancer Hospital, Hangzhou Institute of Medicine (HIM), Chinese Academy of Sciences, Hangzhou, Zhejiang China; 2 The Second Clinical Medical College, Zhejiang Chinese Medical University, Hangzhou, Zhejiang China

**Keywords:** Cancer, Oesophageal cancer, Surgical oncology, Prognostic markers, Risk factors

## Abstract

The C-reactive protein-albumin-lymphocyte (CALLY) index has been identified as a useful and sensitive predictive tool for stratification in cancers. This investigation aimed to validate the prognostic ability of CALLY in esophageal squamous cell carcinoma (ESCC). Clinical characteristics of 318 patients with ESCC who underwent radical excision were gathered and analyzed retrospectively. A restricted cubic spline (RCS) model was used to determine an ideal threshold of CALLY due to the non-linear relation. To investigate the predictors, Cox hazard regression analysis was used. The recursive partitioning analysis (RPA), a method of risk categorization, was also developed for prognostic prediction. The receiver operating characteristic (ROC) curves and decision curve analysis (DCA) curves were used to distinguish from the traditional TNM stage. Patients were compared by groups according to the optimal threshold of CALLY index, which was depicted by the non-linear relation between the cancer-specific survival (CSS) and CALLY index (P < 0.0001). Compared to those with high CALLY index, patients with low CALLY index experienced significantly worse 5-year CSS (21.8% vs. 62.6%, P < 0.001). At different TNM stages, patients with high CALLY index also had better 5-year CSS (I: P = 0.029; II: P < 0.001; III: P < 0.001) in subgroup analyses. The hazard ratio for CSS was 0.368 and CALLY index was an independent predictive factor (P < 0.001). Using TNM stage and CALLY-based RPA algorithms, a new staging was created. The RPA model considerably outperformed the TNM classification for prognostication using ROC (P < 0.001). The DCA also demonstrated that the new model outperformed the TNM stage with significantly improved accuracy for CSS. The prognostic value of CALLY in ESCC undergoing radical resection was initially determined in this study. CALLY was substantially related to prognosis and might be utilized in conjunction with TNM to evaluate ESCC prior to surgery.

## Introduction

Within the gastrointestinal malignancies in the world, esophageal cancer (EC) is one of the leading aggressive forms^1^. Adenocarcinoma and squamous cell carcinoma (SCC), which is still the most common kind of EC in China, are the two primary histologic subtypes of the disease^2^. Despite the medical and technological advances and improved comprehensive therapeutic methods in recent years, the prognosis of EC is still unsatisfactory, posing a significant threat to human health^3^. Thus, better assessment of recurrence and survival risk in EC is critically essential for clinical decision‐making.

It is noted that inflammatory-immune responses have a crucial role in cancer initiation, progression and metastasis^4^. Therefore, various hematological indices that reflect immune function and inflammatory response, such as platelet (PLT) to lymphocyte (LYM) ratio (PLR), LYM to monocyte (MON) ratio (LMR), neutrophil (NEU) to LYM ratio (NLR), systemic immune inflammation index (SII), and systemic inflammatory response index (SIRI), are crucial for the prognosis of a variety of cancers^5–8^. Moreover, accumulating evidence demonstrates that several hematological indices representing the host nutritional status, such as albumin (ALB) and C-reactive protein to ALB ratio (CAR), are closely related to prognosis^9,10^. However, these indices alone may not provide sufficient values due to their own limitations. Therefore, it is urgent to find an effective index that comprehensively reflects the nutritional status, inflammatory level and immune function to better predict prognosis.

It is established that hematological indices are usually used to reflect the nutritional status immune function and inflammatory level in cancer patients^11^. Increases in the knowledge of inflammation, nutrition and immune reactions have demonstrated that these parameters might take additional prognostic information in cancer. The status of inflammation, nutrition, and immune function are reflected in the unique CRP-albumin-lymphocyte (CALLY) index, which has recently been established and proved to be a better indicator of hepatocellular carcinoma^12^. After that, the CALLY has been widely used in oral cavity cancer, colorectal cancer, cholangiocarcinoma, and epithelial ovarian cancer^13–16^.

However, the clinical utility of CALLY in esophageal SCC (ESCC) remains unclear. This study therefore set out to investigate the preoperative CALLY index in ESCC with radical resection in order to better understand its predictive significance. Moreover, the prognostic values between CALLY and other classical indices were compared to determine the superiority. In addition, a new staging based on CALLY index was also created and exhibited significantly superior performance for prognosis.

## Materials and methods

### Study design and patient selection

**Figure 1:**
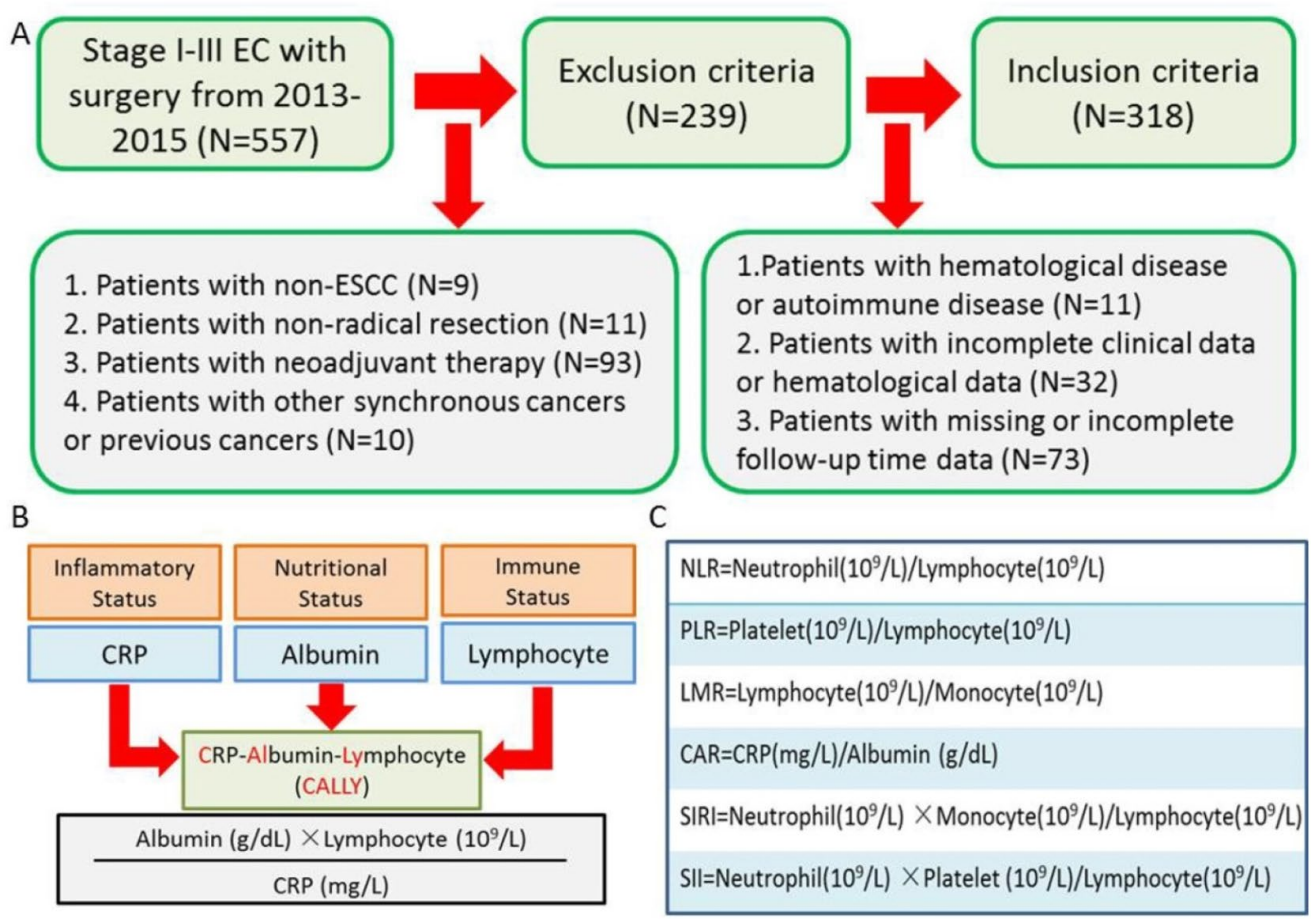
The inclusion and exclusion criteria (**A**). The definitions of CALLY (**B**) and other indices (**C**).

Retrospective data in patients with ESCC who underwent radical excision at our department between Jan. 2013 and Dec. 2015 was gathered and analyzed. Patients who underwent radical resection for thoracic TNM stage I-III ESCC were included. Before surgery, patients did not receive any neoadjuvant therapy (NAT) or further anticancer treatment. Patients with any hematologic, inflammatory, or autoimmune diseases were excluded. Patients with any malignancies that coexisted or had previously been diagnosed were also excluded. Figure 1A depicted the patient selection flowchart. In compliance with the Helsinki Declaration, this study was carried out. Due to the retrospective character, the Zhejiang Cancer Hospital's ethical committee approved this study (IRB-2021-4) and waived informed consent.

### Therapy and follow-up

According to prior reports, the study's surgical techniques included either the Ivor Lewis or McKeown procedure, which involved a subtotal minimally or open invasive esophagectomy in addition to a two-field lymphadenectomy^17^. Because of the potential impact of NATs on preoperative hematological indices, patients having NATs were excluded from the study. Adjuvant therapy is controversial, and it is generally believed that adjuvant therapy, such as chemotherapy or radiochemotherapy, was required for those with T3-4 and/or N1-3^18,19^. After the end of treatment, patients were then subsequently followed up on at regular intervals. The final instance was finished in December 2020.

### Gathering and defining data

Retrospective data extraction from the medical records revealed the demographic features and the clinicopathological characteristics. The tumor stage in the current study used the 8th AJCC/UICC TNM classification^20^. A week prior to surgery, preoperative laboratory indices, such as NEUs, LYMs, MONs, PLTs, ALBs, and CRP, were also acquired via biochemical and blood routine tests. The CALLY index was determined as previously explained^12–16^. Figure 1B displayed an overview of the CALLY calculation. The definitions of other indices were displayed in Fig. 1C based on previously published studies^5–10^.

### Statistical analysis

For continuous data, Mann–Whitney u-tests or Student's t-tests were used, whilst Fisher's exact tests or Chi-square were used for categorical data. CALLY and other laboratory indices' areas under the curves (AUCs) were compared using receiver operating characteristic (ROC) curves. Clinical applicability was also compared using decision curve analysis (DCA) curves. A restricted cubic spline (RCS) model was also used to determine the ideal threshold for CALLY index in accordance with the analysis of the non-linear relation between ESCC prognosis and CALLY. Predictors in hazard ratios (HRs) and 95% confidence intervals (CIs) for cancer-specific survival (CSS) were found using Cox regress analyses. Kaplan–Meier curves were also used to compare the survival differences. Recursive partitioning analysis (RPA) was carried out to construct a risk stratification model that included CALLY index and TNM for prognostication and stratification. The DCA and ROC curves were used to assess the prognostic performance of the RPA-based model. With the help of R 4.1.2, SPSS 20.0, and Medcalc 17.6, statistical analyses were conducted. Statistical significance was defined as a p value < 0.05.

### Ethics approval and consent to participate

This study was performed in accordance with the Declaration of Helsinki and approved by the Ethics Committee of Zhejiang Cancer Hospital (IRB-2021-4).

## Results

### Patient characteristics

**Figure 2:**
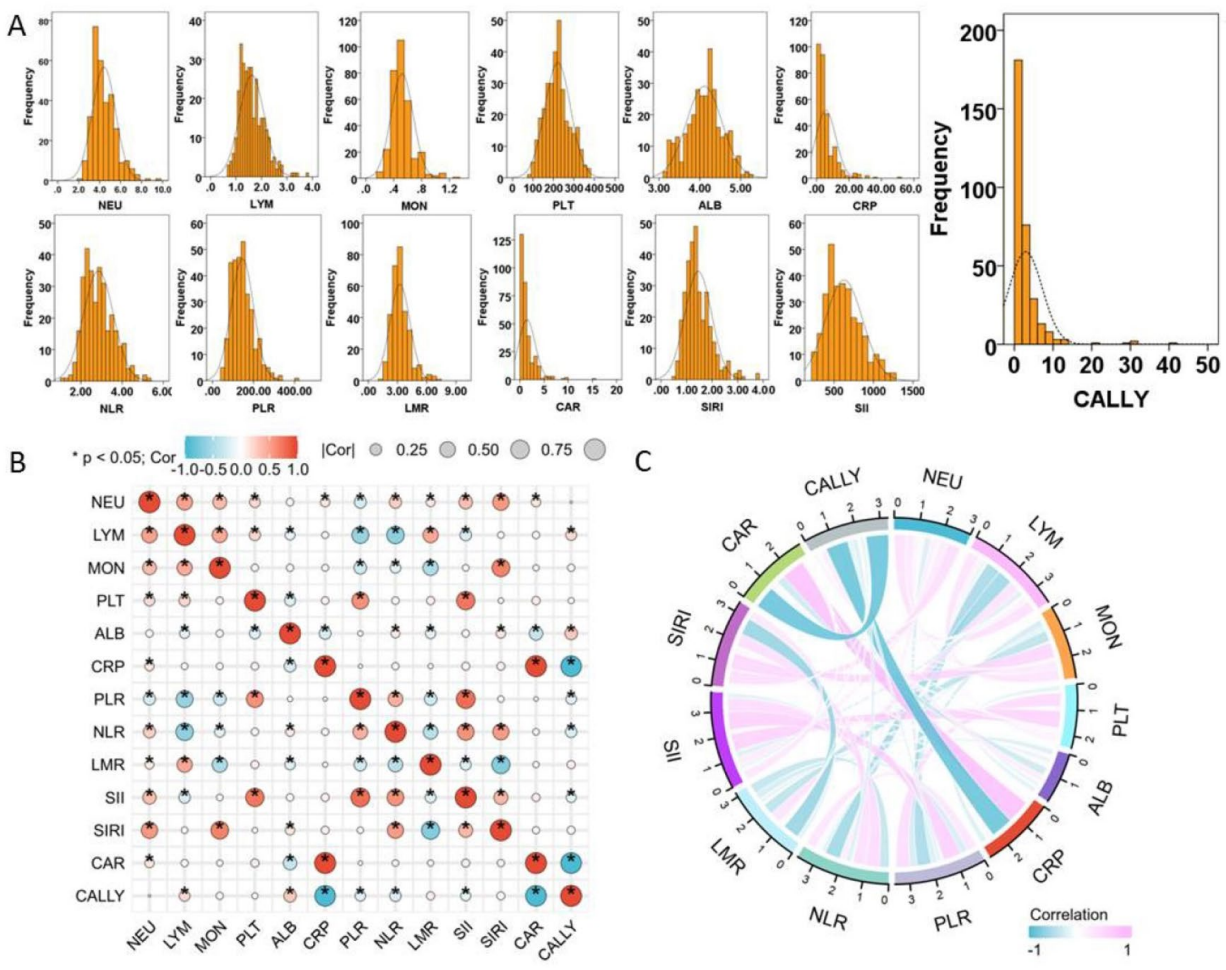
The distribution of CALLY and other indices (**A**). The correlation heatmap (**B**) and chord diagram (**C**) for all indices.

Table 1 displayed the hematological indices and patient characteristics. In this study, 318 patients with a mean age of 59.4 ± 7.1 years (range: 39–76 years) who had radical resection of ESCC were included. The male to female ratio among all patients was 2.03:1, with 105 (33.1%) females and 213 (67.0%) males. According to the TNM classification, there were 110 (34.6%), 93 (29.2%) and 105 (36.2%) patients in stage I, II and III, respectively. Within the study, the follow-up period ranged from 8 to 94 months, with an average of 47 months. The mean value of CALLY index was 2.92 ± 4.30 (range: 0.10–40.85). The distribution of CALLY and other indices is shown in Fig. 2A. Figure 2B,C displayed the correlation and chord diagrams for all hematological indices.

**Table 1:** Clinical and hematological characteristics of ESCC.

	Total (n = 318)
Age (mean ± SD, years, range)	59.4 ± 7.1 (39–76)
Sex (female/male, n, %)	105 (33.0)/213 (67.0)
Tumor location (upper/middle/lower, n, %)	22 (6.9)/142 (44.7)/154 (48.4)
Differentiation (well/moderate/poor, n, %)	51 (16.0)/207 (65.1)/60 (18.9)
Vessel invasion (yes/no, n, %)	54 (17.0)/264 (83.0)
Perineural invasion (yes/no, n, %)	62 (19.5)/256 (80.5)
Tumor length (mean ± SD, cm, range)	4.03 ± 1.87 (0.5–13.5)
TNM stage (I/II/III, n, %)	110 (34.6)/93 (29.2)/115 (36.2)
Adjuvant therapy (yes/no, n, %)	95 (29.9)/223 (70.1)
Neutrophil (mean ± SD, 10^9^/L, range)	4.41 ± 1.12 (2.1–9.7)
Lymphocyte (mean ± SD, 10^9^/L, range)	1.62 ± 0.49 (0.7–3.8)
Monocyte (mean ± SD, 10^9^/L, range)	0.52 ± 0.16 (0.2–1.3)
Platelet (mean ± SD, 10^9^/L, range)	220.7 ± 63.0 (86–503)
Albumin (mean ± SD, mg/L, range)	4.11 ± 0.44 (3.15–5.21)
C-reactive protein (mean ± SD, g/L, range)	5.71 ± 6.15 (0.2–52.2)
NLR (mean ± SD, range)	2.85 ± 0.73 (1.12–5.33)
PLR (mean ± SD, range)	146.0 ± 54.6 (50.0–419.2)
LMR (mean ± SD, range)	3.25 ± 1.03 (1.40–11.33)
CAR (mean ± SD, range)	1.45 ± 1.69 (0.04–15.54)
SIRI (mean ± SD, range)	1.46 ± 0.52 (0.57–3.82)
SII (mean ± SD, range)	626.2 ± 236.0 (218–1761)
CALLY (mean ± SD, range)	2.92 ± 4.30 (0.10–40.85)

ESCC: esophageal squamous cell carcinoma; SD: standard deviation; TNM: tumor node metastasis; NLR: neutrophil to lymphocyte ratio; PLR: platelet to lymphocyte ratio; LMR: lymphocyte to monocyte ratio; CAR: C-reactive protein to albumin ratio; SIRI: systemic inflammatory response index; SII: systemic immune-inflammation index; CALLY: C-reactive protein-albumin-lymphocyte.

### Prognostic comparison between CALLY and other indices

**Figure 3:**
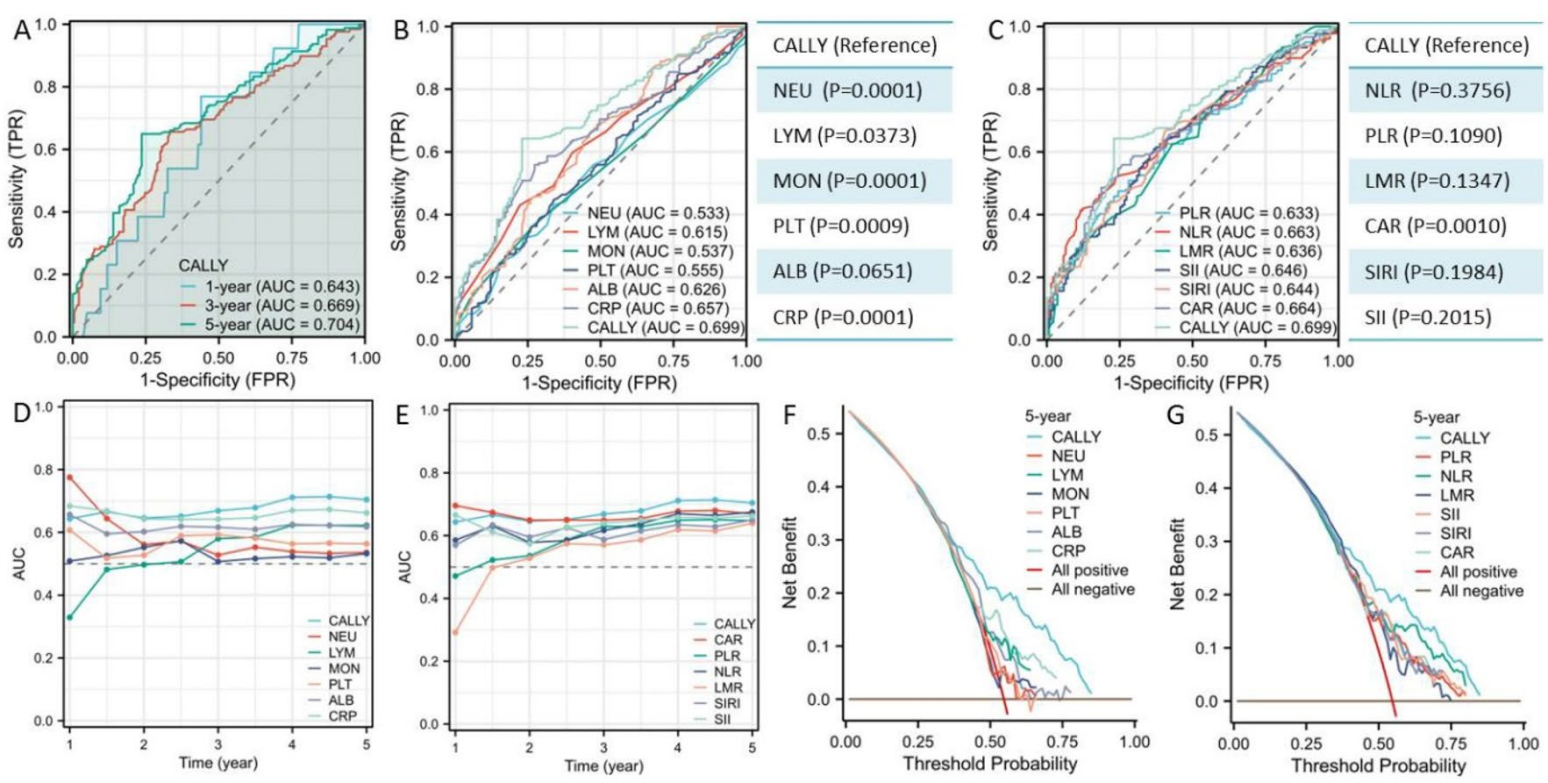
The CSS prediction based on CALLY by ROC (**A**). ROC comparisons between CALLY and single indices (**B**) and other conventional indices (**C**). Time-dependent ROC comparisons between CALLY and single indices (**D**) and other conventional indices (**E**). Comparisons by DCA between CALLY and single indices (**F**) and other conventional indices (**G**).

The CSS prediction based on CALLY were shown in Fig. 3A. Based on the ROC curves, the AUCs of CALLY for 1-, 3- and 5-year CSS prediction were 0.643, 0.669 and 0.704, respectively. To better understand the prognostic value of CALLY, ROC analyses between CALLY and other conventional indices (SIRI, LMR, PLR, NLR, CAR, and SII) and single indices (LYM, MON, PLT, NEU, CRP and ALB) were performed. According to the ROC curves, CALLY had the highest AUC (0.699) in comparison to the other indices, demonstrating CALLY's superior predictive capacity (Fig. 3B,C). In Fig. 3D,E, the time-dependent ROC curves were also displayed and discovered CALLY’s greater predictive value. The DCA curves further supported CALLY's superior predictive value when compared to other indices (Fig. 3F,G).

### Patient characteristics grouped by CALLY index

**Figure 4:**
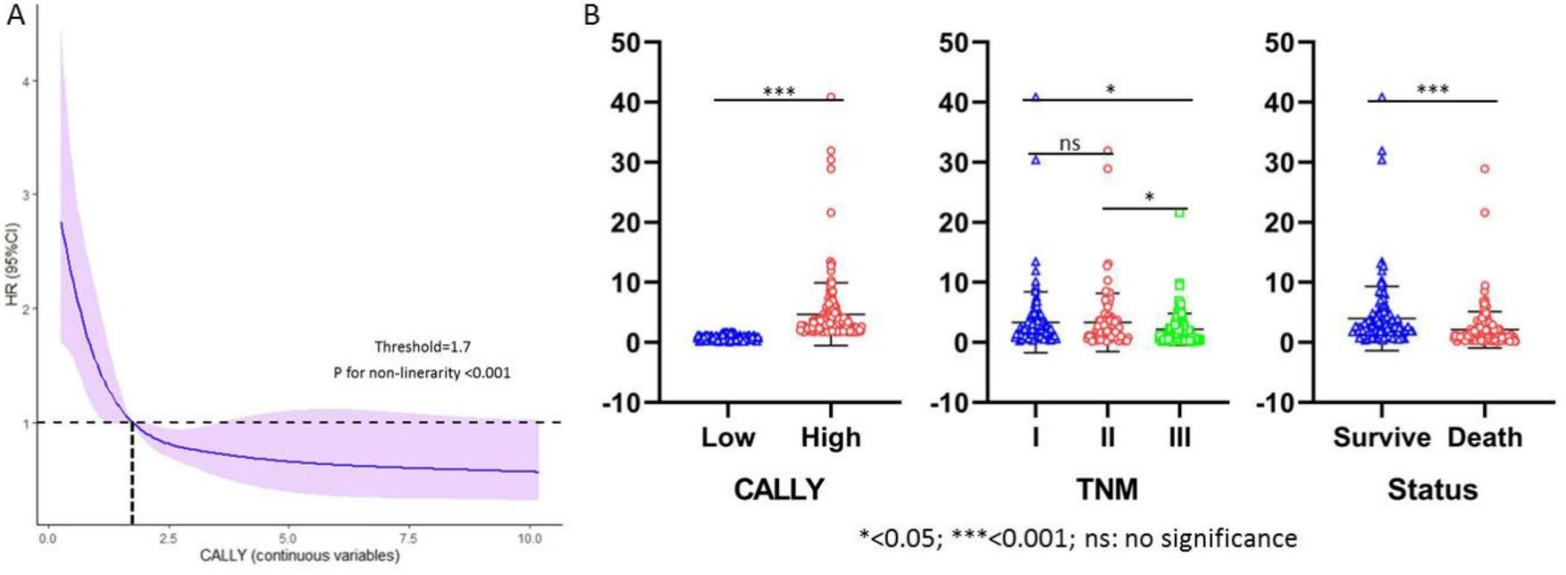
The optimal cut-off value of CALLY based on the RCS (**A**). The violin plots regarding CALLY, TNM stage and CSS (**B**).

The increasing non-linear association between CALLY and CSS was depicted using the RCS analyses with five knots (P < 0.0001). Once the appropriate threshold of 1.7 for CALLY index was determined, patients were separately split into two groups (Fig. 4A). Patients with low CALLY also had a considerably higher tumor burden and mortality risk according to the scatter plots (Fig. 4B). Table 2 displays the clinical characteristics that were compared. Patients with high CALLY index had, accordingly, lower rates of perineural and/or vascular invasion (P = 0.035), lower tumor lengths (P = 0.020), and earlier TNM stages (P < 0.001).

**Table 2:** Patient characteristics grouped by CALLY in ESCC.

	Low-CALLY (n = 147)	High-CALLY (n = 171)	P-value
Age (≤ 60/ > 60, years, n)	93 (63.3)/54 (36.7)	94 (55.0)/77 (45.0)	0.134
Sex (female/male, n)	47 (32.0)/100 (68.0)	58 (33.9)/113 (66.1)	0.713
Tumor location (n)			0.601
Upper	8 (5.4)	14 (8.2)	
Middle	68 (46.3)	74 (43.3)	
Lower	71 (48.3)	83 (48.5)	
Differentiation (n)			0.283
Well	21 (14.3)	30 (17.5)	
Moderate	93 (63.3)	114 (66.7)	
Poor	33 (22.4)	27 (15.8)	
Vessel invasion (yes/no, n)	32 (21.8)/115 (78.2)	22 (12.9)/149 (87.1)	0.035
Perineural invasion (yes/no, n)	39 (26.5)/108 (73.5)	23 (13.5)/148 (86.5)	0.003
Tumor length (≤ 3.0/ > 3.0, n)	41 (27.9)/106 (72.1)	69 (40.4)/102 (59.6)	0.020
TNM stage (n)			0.007
I	39 (26.5)	71 (41.6)	
II	43 (29.3)	50 (29.2)	
III	65 (44.2)	50 (29.2)	
Adjuvant therapy (yes/no, n)	47 (32.0)/100 (68.0)	48 (28.1)/123 (71.9)	0.448

CALLY: C-reactive protein-albumin-lymphocyte; ESCC: esophageal squamous cell carcinoma; SD: standard deviation; TNM: tumor node metastasis.

### CSS Kaplan–Meier curves and analysis by subgroup

**Figure 5:**
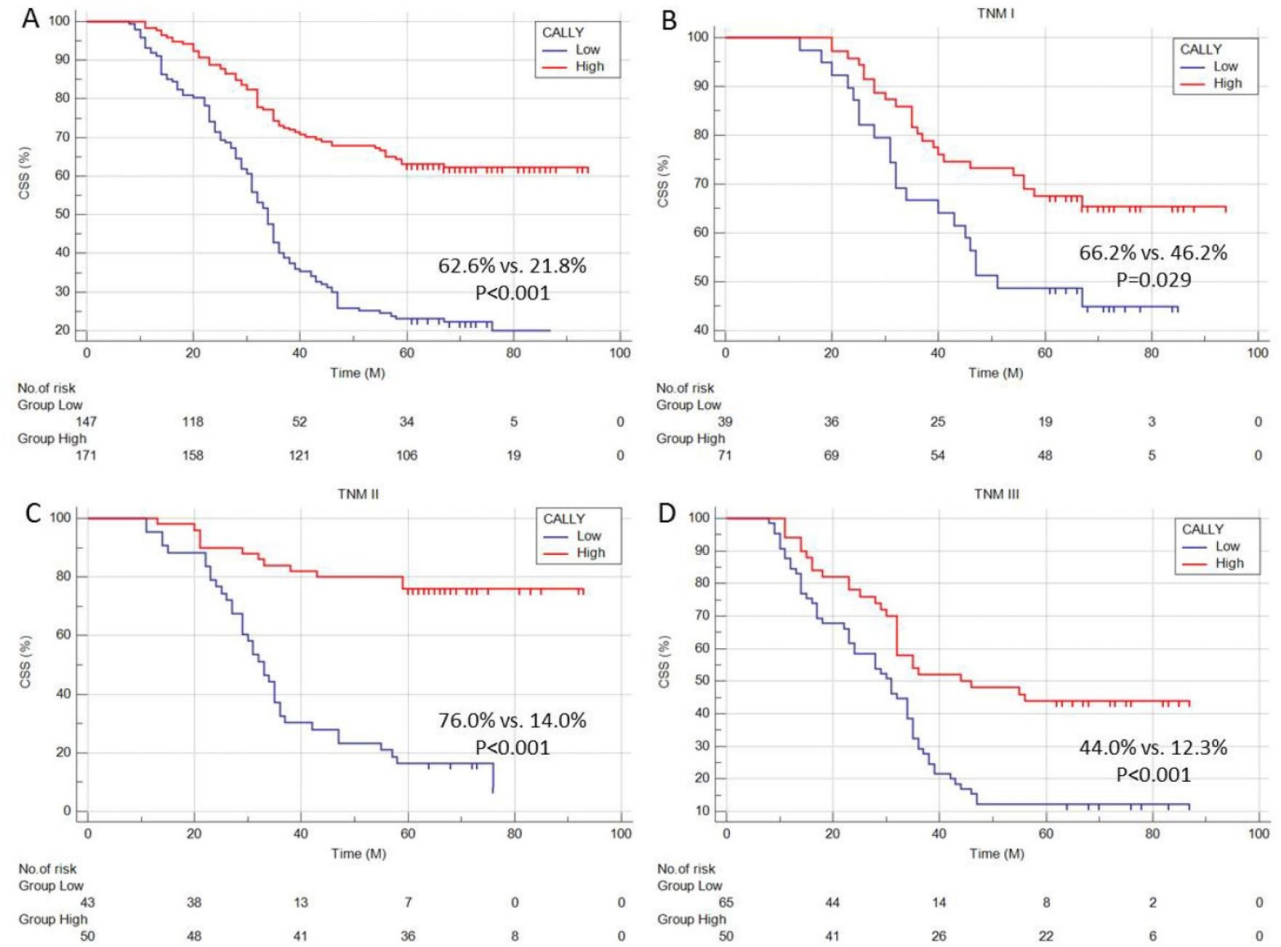
The survival curves of CSS grouped by CALLY (**A**). Subgroup analyses grouped by CALLY in TNM I (**B**), TNM II (**C**) and TNM III (**D**).

Figure 5A displayed the CSS curves grouped by CALLY index. Patients with high CALLY had significantly better 5-year CSS (62.6% vs. 21.8%, P < 0.001) than patients in the low group. To validate the 5-year CSS’s statistically significant differences at different TNM stages in ESCC, subgroup analyses were also carried out and confirmed the prognostic stratification (TNM I: 66.2% vs. 46.2%, P = 0.029; TNM II: 76.0% vs. 14.0%, P < 0.001; TNM III: 44.0% vs. 12.3%, P < 0.001; Fig. 5B–D). This made it possible for researchers to comprehend the influence of the CALLY index on prognosis in various TNM stages.

### Independent prognostic factors in Cox regression analyses

Predictors in HRs and 95% CIs for CSS were found using Cox regress analyses. CALLY index (P < 0.001), TNM stage (P < 0.001), tumor length (P = 0.038), vascular invasion (P = 0.002), and perineural invasion (P = 0.001), which were all significant predictive indices in the univariate Cox analyses for CSS, were enlisted for subsequent analyses (Table 3). The CALLY index had a statistically significant relationship with CSS and could be utilized as a standalone and effective prognostic indicator (HR = 0.368, 95% CI: 0.268–0.506, P < 0.001).

**Table 3:** Cox analyses of prognostic factors associated with CSS in ESCC.

	Univariate analysis		Multivariate analysis	
	HR (95% CI)	P value	HR (95% CI)	P value
Age (years, > 60 vs. ≤ 60)	0.792 (0.585–1.071)	0.130		
Sex (male vs. female)	0.831 (0.611–1.129)	0.236		
Tumor location		0.583		
Upper	Reference			
Middle	1.321 (0.705–2.478)	0.385		
Lower	1.175 (0.627–2.203)	0.615		
Differentiation		0.042		0.157
Well	Reference		Reference	
Moderate	1.244 (0.801–1.931)	0.331	1.039 (0.657–1.642)	0.871
Poor	1.819 (1.096–3.019)	0.021	1.467 (0.867–2.485)	0.154
Vessel invasion (yes vs. no)	1.596 (1.111–2.293)	0.011	1.141 (0.774–1.682)	0.505
Perineural invasion (yes vs. no)	1.806 (1.291–2.527)	0.001	1.254 (0.871–1.804)	0.223
Tumor length (cm, > 3 vs. ≤ 3)	1.320 (0.963–1.808)	0.084		
Adjuvant therapy (yes vs. no)	1.011 (0.733–1.393)	0.984		
TNM stage		< 0.001		< 0.001
I	Reference		Reference	
II	1.466 (0.978–2.197)	0.064	1.464 (0.972–2.206)	0.068
III	2.745 (1.910–3.947)	< 0.001	2.253 (1.542–3.292)	< 0.001
CALLY (high vs. low)	0.322 (0.237–0.439)	< 0.001	0.368 (0.268–0.506)	< 0.001

CSS: cancer-specific survival; ESCC: esophageal squamous cell carcinoma; HR: hazard ratio; CI: confidence interval; TNM: tumor node metastasis; CALLY: C-reactive protein-albumin-lymphocyte.

### A risk stratification model created by CALLY and TNM

**Figure 6:**
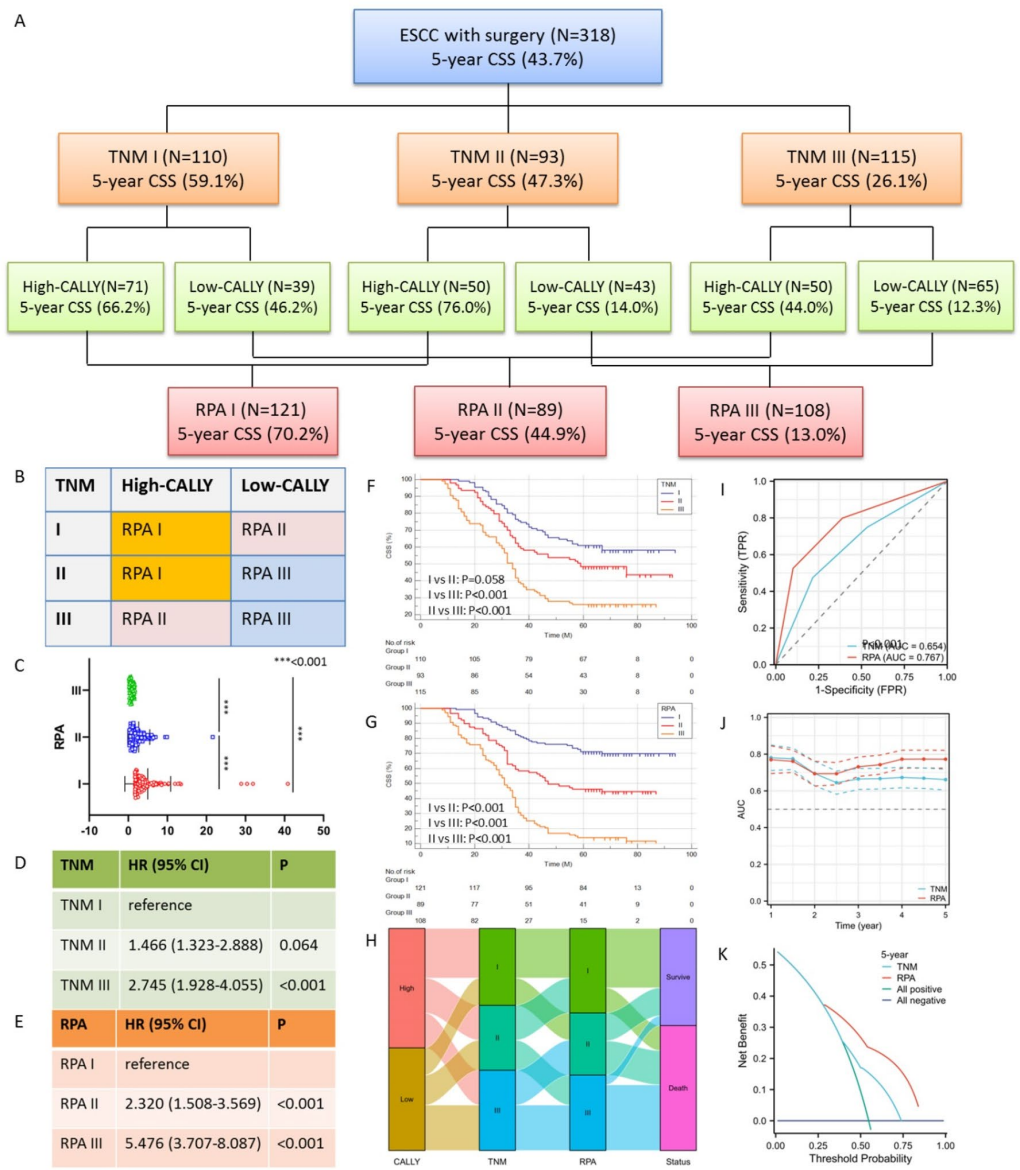
Risk groups derived using RPA classification system combining TNM and CALLY (**A**). The RPA divided into three groups (**B**). Scatter plots grouped by RPA (**C**). Prognostic performances of the TNM (**D**) and RPA (**E**). Kaplan–Meier curves for CSS of TNM (**F**) and RPA risk classifications (**G**). The Sankey diagram regarding CALLY, TNM stage, RPA model and CSS (**H**). ROC (**I**), real-time AUC (**J**) and DCA (**K**) comparing the prognostic performance of the proposed RPA classification system against the TNM stage.

To carry out a new staging, the RPA algorithm using TNM stage and CALLY was used (Fig. 6A). RPA I (n = 121), II (n = 89), and III (n = 108) were the three groups into which the 318 cases were separated (Fig. 6B). Patients grouped by the RPA-based model had a considerably significance according to the scatter plots (Fig. 6C). In terms of HR, the RPA-based model outperformed the TNM classification when tested for hazard discrimination (Fig. 6D,E). Better stratification was also shown by the RPA-based model according to the CSS curves (Fig. 6F,G). The Sankey diagram regarding CALLY, TNM stage, RPA model and CSS is shown in Fig. 6H. The RPA model's prediction capabilities were then evaluated in comparison to TNM classification. The results indicated that the RPA's predictive accuracy was considerably better than that of TNM classification (AUC_RPA_ = 0.716 and AUC_TNM_ = 0.663, P = 0.045; Fig. 6I). The RPA-based model's superior prognostic accuracy was also validated by the teal-time AUCs (Fig. 6J) and DCAs (Fig. 6K).

## Discussion

At present, the AJCC TNM classification is the most commonly used tool in ESCC to stratify patients, optimize treatments and predict survival. However, a limitation of the above TNM system is that it only considers the characteristics of cancer, ignoring host elements that may affect cancer prognosis, such as nutritional status, immune function and inflammatory status^21^. Several nutritional and immune-inflammatory related parameters, such as ALB, CRP and LYM, have been used to predict ESCC prognosis^6,9,10^. However, a single parameter has certain limitations in independently predicting the prognosis of cancer patients. Thus, composite parameters for prognosis prediction are necessary in further studies. Recently, a novel proposed CALLY, combining indices of the nutrition (ALB) and immune-inflammatory response (CRP and LYM), demonstrated a better prognostic discrimination in hepatocellular carcinoma^12^. However, relevant results of CALLY in ESCC have been lacking. In addition, it is uncertain whether CALLY is superior to its components and other conventional indices in determining the prognosis in ESCC.

The relationships between preoperative CALLY and ESCC results were examined in this study. Consistent with the previous research on the association between cancer-related nutritional status and immune-inflammatory response and cancer metastasis, the results demonstrated that patients with higher tumor stages and worse prognoses had lower CALLY values^22^. The prognostic roles between CALLY and other classical laboratorial indices were compared to determine the clinical superiority. Notably, CALLY demonstrated the highest predictive capacity in terms of CSS in DCA and ROC analyses among all the most often used indices. As a result, CALLY emerged as the top potential index for prognostic classification in ESCC based on nutrition and immune-inflammatory factors. Finally, using the RPA algorithm along with the TNM and CALLY index, a new staging was established. The model exhibited noticeably better prognostication performance when compared to the TNM classification.

When the CALLY index was initially introduced in hepatocellular carcinoma, it was found to be much stronger than other conventional indices and was closely connected with cancer prognosis^12^. After that, CALLY's prognostic usefulness in a number of malignancies was verified^13–16^. A study including 279 oral cavity cancer patients with surgery revealed that preoperative CALLY is a simple and inexpensive prognostic score^13^. Another investigation study including 1260 colorectal cancer cases reported that CALLY index was independently related to prognosis and had a higher clinical value^14^. Moreover, CALLY index was also significantly related to long-term outcomes in cholangiocarcinoma after surgery, suggesting the significant importance of comprehensive assessment of nutritional status and immune inflammatory response^15^. In addition, with an optimal cut-off value of 3.0, high CALLY index was related to better survival outcomes in those with epithelial ovarian cancer^16^. Although the CALLY was significantly related to prognosis in various cancers, the cut-off value of CALLY was different. In terms of the non-linear relationships between CALLY and prognosis, the best threshold of CALLY in our study was computed using the RCS. CALLY demonstrated the highest predictive capability for CSS among all the most popular indices. As a result, CALLY was chosen as the best potential indicator for stratification of nutritional and immune-inflammatory prognosis.

According to reports, the nutritional status and immune-inflammatory response as well as cancer behavior are all linked to cancer prognosis^23,24^. As is well known, ALB has been shown to predict the nutritional status and prognosis of cancer patients^25^. Tumor necrosis factor (TNF)-induced increased microvasculature permeability and interleukin-1 (IL-1) and interleukin-6 (IL-6) induced inhibition of albumin synthesis significantly decreased serum ALB levels in cancers^26^. Serum CRP can cause systemic inflammation by secreting various pro-inflammatory cytokines, such as IL-1, IL-6 and TNF-α, resulting in the gradual loss of important protein components in host, leading to the death of cancer patients^27^. Study has demonstrated a substantial correlation between elevated CRP levels and a higher TNM stage for cancer as well as a bigger inflammatory response^28^. LYMs are widely used as a measure of immunological competence. LYMs also have the ability to enter the tumor microenvironment and disrupt the growth and spread of tumor cells. On the other hand, LYMs have a role in immune modulation inside the tumor microenvironment, helping to mount an effective defense against tumor cells^29,30^. Because the CALLY is associated with tumor-related factors as well as nutritional and immune-inflammatory status, it may be a better prognostic index than other conventional indices.

Some of the strengths of this study should be acknowledged. Firstly, it is for the first time to assess the predictive role of CALLY index in ESCC. The findings showed that ESCC patients with a larger tumor stage and a worse prognosis had a lower baseline value of CALLY index. Secondly, the prognostic roles between CALLY and other classical indices were compared in order to determine the clinical superiority of CALLY index. Notably, CALLY demonstrated the strongest predictive capability among all widely used indices in terms of CSS in ESCC. Thirdly, the CALLY index may have high practicability in the daily clinical practice of ESCC due to the simple, convenient and inexpensive calculating from the routine laboratory tests. Fourthly, patients with low CALLY index may have nutritional and immune-inflammatory conditions that promote tumor growth, thereby making TNM stage predictive. In order to undertake a new staging, the RPA algorithm using TNM stage and CALLY index was used. The accuracy for predicting survival was greatly increased, and the RPA risk categories performed better than the well-established TNM stage.

This study's limitations should be taken into account. To start with, bias was unavoidable because this research was done in a single center and was retrospective in nature. Second, CALLY may be impacted in a variety of circumstances because it is a straightforward and innovative index generated from peripheral blood. As a result, CALLY's application can be restricted. Thirdly, because patients who got NAT were excluded from the present study, the findings might be restricted. Fourthly, the current study lacked the associations between postoperative level of CALLY and prognosis. The prognostic benefits of postoperative CALLY index still need further study. Therefore, the prognostic validity of CALLY index needs to be confirmed with other perspective researches.

## Conclusion

In ESCC patients who underwent radical resection, the simple and novel prognostic score CALLY index was confirmed. Due to the close relationship between the tumor stage and prognosis and CALLY index, ESCC patients might use it for preoperative evaluation. Clinicians may benefit from using the RPA stratification model based on CALLY index to provide individualized prognostication.

## Data Availability

All data are available upon request. Further inquiries can be directed to the corresponding author.
